# Renovascular involvement of systemic vascular disease: a pictorial review

**DOI:** 10.1007/s00261-022-03591-5

**Published:** 2022-07-07

**Authors:** Yousuf Qaseem, Fiona Cassidy, Lejla Aganovic, Andrei Purysko, Sara Mirza, Noushin Vahdat

**Affiliations:** 1 Department of Radiology, University of California San Diego Health, San Diego, CA USA; 2 Department of Radiology, Veteran Administration Healthcare System, San Diego, CA USA; 3 Department of Radiology, Cleveland Clinic Foundation, Cleveland, OH USA

**Keywords:** Kidney, Vasculitis, Vascular diseases, Cardiovascular diseases, Autoimmune diseases

## Abstract

**Abstract:**

Like many solid organs, the kidneys are susceptible to a wide variety of systemic vascular diseases. Comprising a significant subset of these diseases are the vasculitides, broadly encompassing numerous inflammatory conditions of the blood vessels. However, many of these conditions are non-vasculitic and non-inflammatory, and differentiation of these entities is crucial to guide the initiation of proper therapy. These non-vasculitic diseases include coagulopathic conditions leading to vascular complications, hemolysis, and hematogenous processes that can affect multiple organ systems. These systemic diseases can result in both macrovascular and microvascular pathology, involving the arteries, veins, and smaller vessels, and management of these conditions can differ significantly depending upon the underlying pathophysiology. Because the clinical manifestations of these disease processes can be heterogeneous, ranging from renal dysfunction to life-threatening hemorrhage, proper recognition of these entities is essential to help guide clinicians to the correct diagnosis and prevent potentially disastrous complications. Many of these systemic vascular processes can be detected by non-invasive imaging, including computed tomography (CT) and magnetic resonance imaging (MRI), and identification of their characteristic renal manifestations by radiologists is a critical component of patient care. This review covers a variety of these diseases and their imaging manifestations, to aid in their recognition and better equip radiologists to provide vital diagnostic information that can optimize patient care.

**Graphical abstract:**

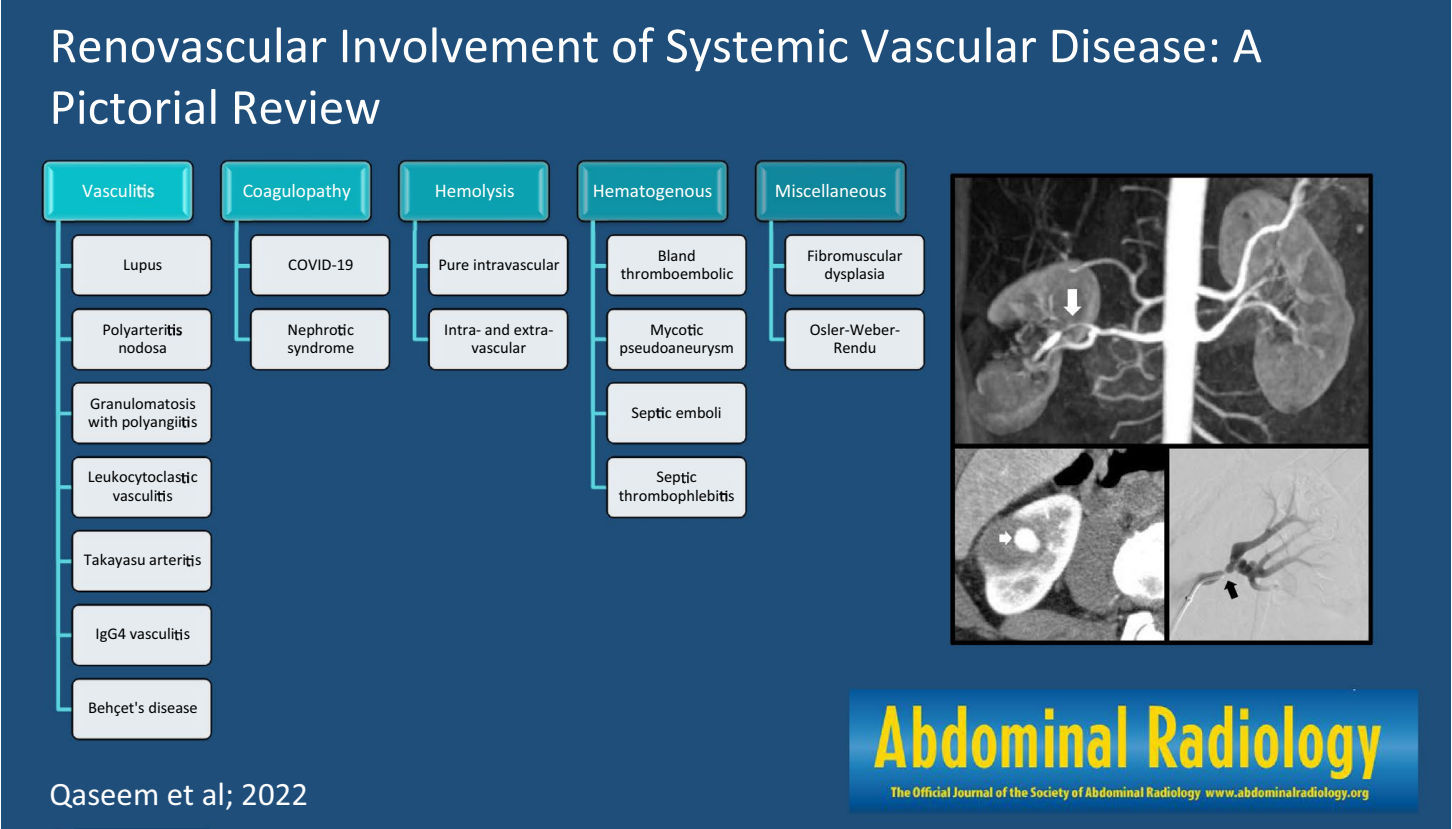

## Introduction

A wide variety of systemic vascular diseases may present with renal involvement. Among these diseases are various subtypes of vasculitides, a broad term describing inflammation of blood vessels, which can have a heterogeneous pattern of involvement, clinical and radiological manifestations. However, renal involvement of systemic vascular disease is not limited to inflammatory conditions, and a variety of non-vasculitic processes may involve the renal vasculature. Systemic vascular diseases may involve the renal arterial system, venous system, or both, and may manifest on a macrovascular or microvascular level. Progression of these conditions can result in significant renal dysfunction, as well as potentially life-threatening complications such as retroperitoneal hemorrhage.

With advances in both computed tomography (CT) and magnetic resonance imaging (MRI), including improvement in angiographic imaging with these modalities, non-invasive radiologic examinations have become a critical element of diagnosing vascular disease. Many systemic vascular diseases can be detected by non-invasive imaging and display characteristic patterns of involvement, which can provide invaluable information to clinicians that can guide diagnosis and treatment. Therefore, recognition of these diseases on imaging is essential to the practicing radiologist to ensure that clinicians may identify these entities and initiate treatment as early as possible to prevent complications.

## Vasculitides

The vasculitides are a broad category of diseases characterized by inflammation of the blood vessels. Although the underlying etiology of these diseases is not always well understood, inflammatory changes and necrosis of the vessel walls can result in a variety of complications including aneurysm and pseudoaneurysm formation, vessel rupture with the potential for life-threatening hemorrhage, and compromise in the vascular lumen resulting in altered hemodynamics [1, 2]. CT and MR angiography have become important imaging tools due to their non-invasive nature and excellent visualization of the vessel wall and lumen [2]. Recognition of characteristic patterns of vascular involvement can help guide the diagnosis of the underlying vasculitis and ultimately lead to the initiation of appropriate treatment to help reduce the risk of significant complications.

### Systemic lupus erythematosus and drug-induced lupus

**Fig. 1:**
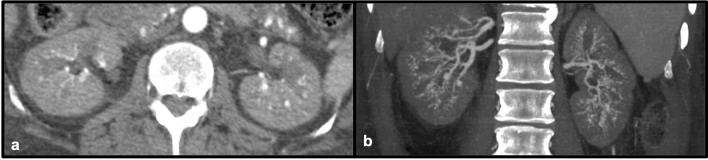
49-year-old male with multiple medical problems found to have laboratory evidence of drug-induced lupus. Axial (**a**) and coronal maximum-intensity projection (MIP) contrast-enhanced CT images (**b**) demonstrate innumerable aneurysms of varying sizes involving several renal artery branches bilaterally. This patient expired 9 days after this CT scan

**Fig. 2:**
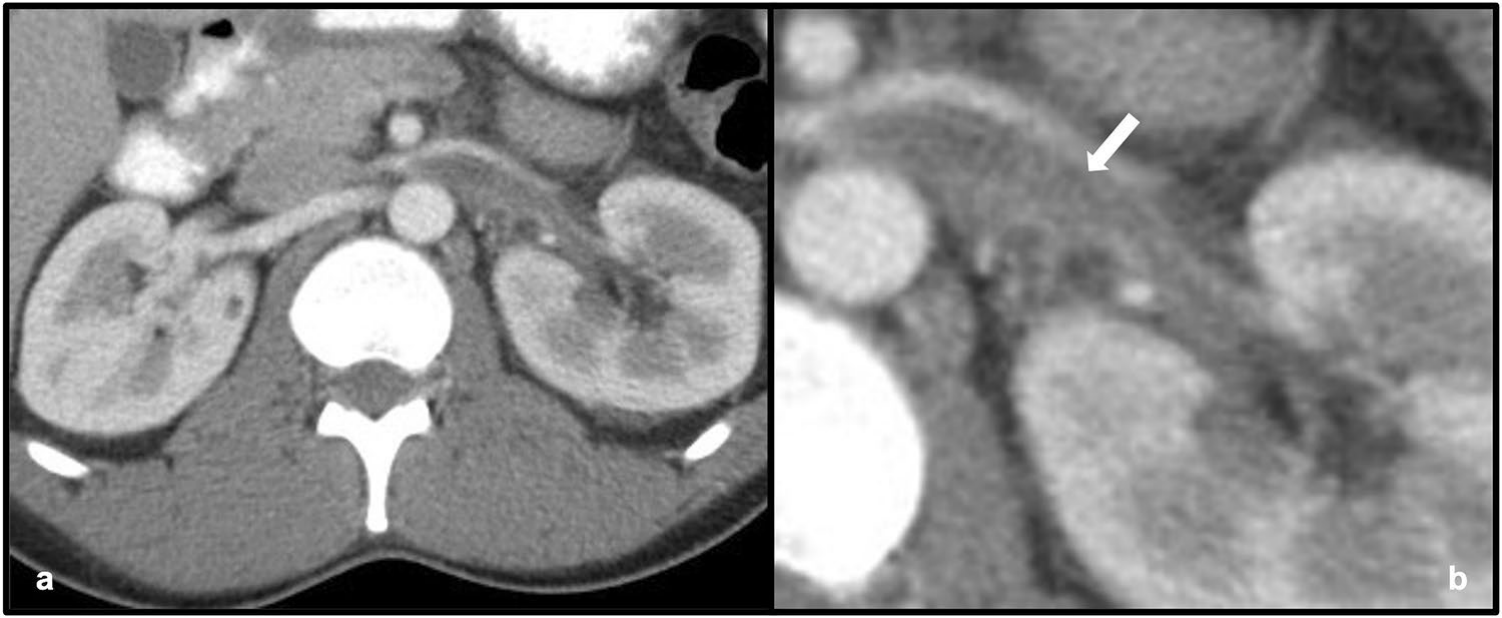
35-year-old male who presented with abdominal pain, found to have lupus nephritis on renal-biopsy. Axial wide (**a**) and narrow (**b**) field-of-view images of a contrast-enhanced CT demonstrate a filling defect within an expanded left renal vein (white arrow), consistent with acute renal vein thrombosis. The normal right renal vein is shown for comparison

Systemic lupus erythematosus (SLE) is a multi-systemic inflammatory process that results in damage to a variety of tissues secondary to antibodies and immune-complex mediated pathways, which can in turn lead to organ dysfunction involving different systems [3]. As with a range of inflammatory conditions, renal involvement is relatively common with SLE. The presence of vasculitis is well described in patients with SLE. While vasculitis in these patients often affects the gastrointestinal tract, it may also involve a variety of other organs [3]. Within the abdominal vasculature, lupus vasculitis may result in the formation of aneurysms which can lead to visceral arterial rupture [3]. These aneurysms may also affect the renal vasculature, and rupture of involved renal vessels leading to perinephric hematoma in the setting of lupus vasculitis is known as Wunderlich syndrome [4]. Lupus may be an idiopathic condition, although in several cases drug-induced lupus has been described. With certain medications, such as hydralazine, a continuum between drug-induced vasculitis and lupus has been proposed [5]. Therefore, recognition of lupus-related complications on imaging is important to help guide clinicians to the correct diagnosis so appropriate treatment can be initiated (Figs. 1 and 2).

### Polyarteritis nodosa

**Fig. 3:**
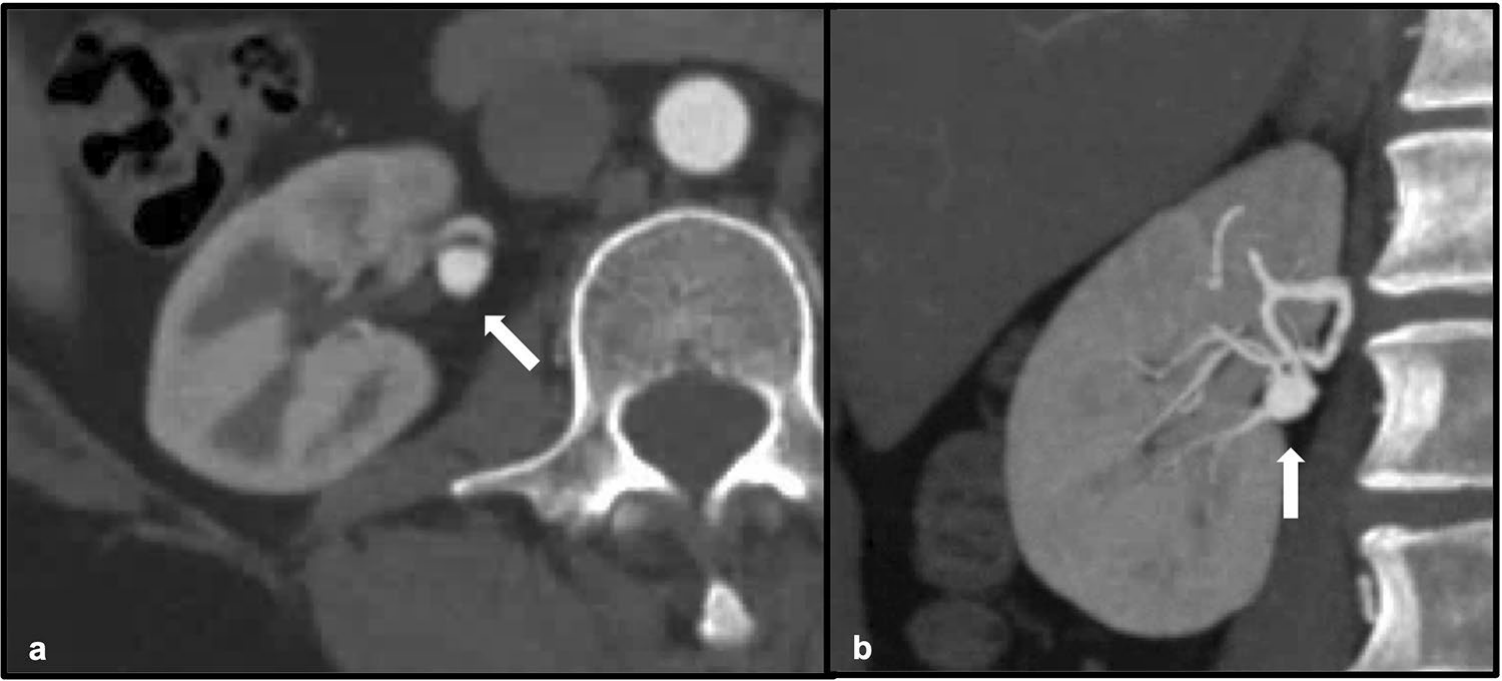
58-year-old female with a history of PAN and report of incidentally noted visceral aneurysms on outside imaging. Axial (**a**) and coronal MIP (**b**) contrast-enhanced CT images demonstrate aneurysmal dilation of a branch of the right renal artery

**Fig. 4:**
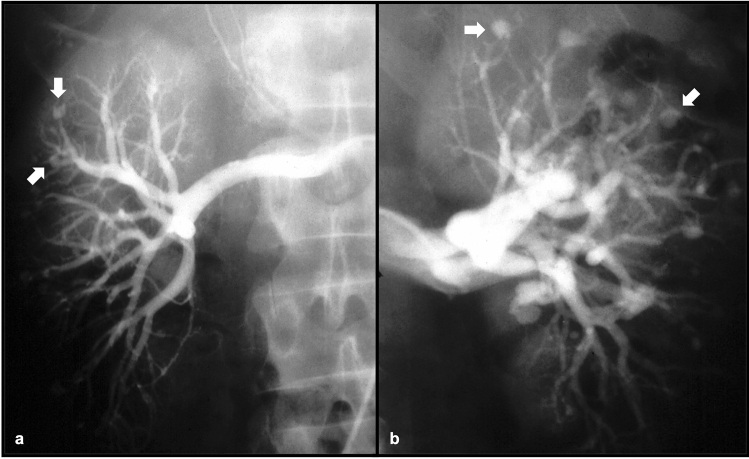
Adult male patient with a history of PAN. Invasive angiographic images of the right (**a**) and left (**b**) kidneys demonstrate classic “micro-aneurysms” (white arrows) of the renal artery branches characteristic of PAN

Polyarteritis nodosa (PAN) is a necrotizing vasculitis that involves small to medium vessels in a variety of organ systems [6]. Involvement of the renal vasculature has been reported in the majority of patients with PAN (ranging from 80–100%) [7]. Because of fibrinoid medial necrosis involving the media within the arcuate arteries, renal infarcts are often reported with PAN, as well as additional findings of aneurysm formation with or without rupture leading to retroperitoneal hemorrhage [8]. While multidetector CT angiography can often demonstrate findings of PAN, conventional angiography is superior at revealing microaneurysms that may be present in the disease process. PAN is typically responsive to therapy with corticosteroids and cyclophosphamide, with remission or cure reported in up to 90% of patients receiving these treatments [8] (Figs. 3 and 4).

### Granulomatosis with polyangiitis

**Fig. 5:**
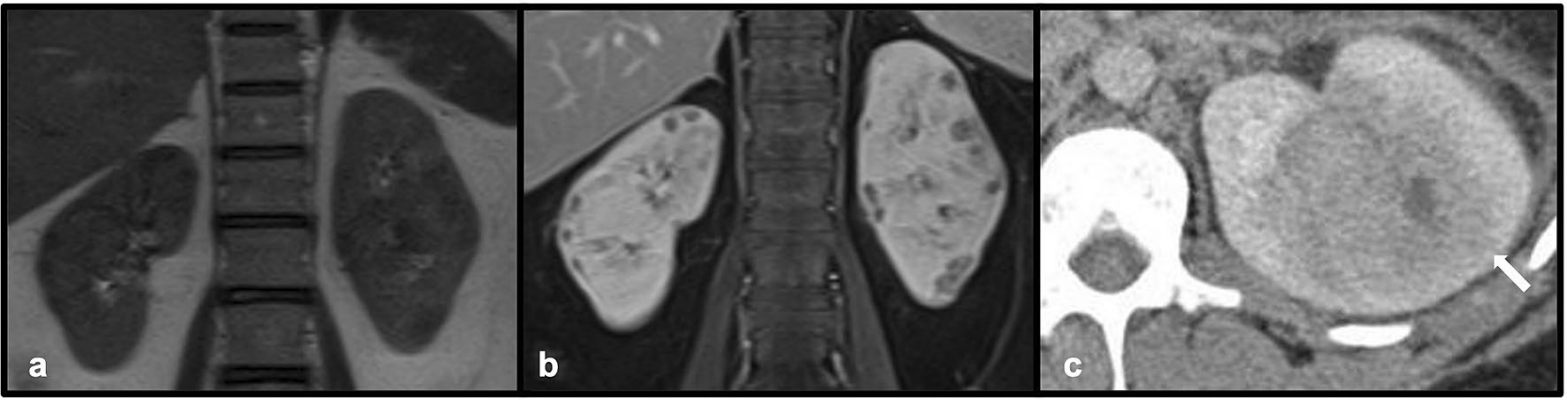
Coronal T2 (**a**) and T1-VIBE post-gadolinium (**b**) images demonstrate numerous small, hypointense bilateral renal cortical defects in a patient with renal-biopsy-proven GPA. Axial post-contrast CT( **c**) in another patient demonstrates a heterogeneous mass in the left kidney with central areas of low attenuation (white arrow) suggestive of necrosis. Biopsy confirmed the presence of non-caseating granulomas consistent with GPA

Granulomatosis with polyangiitis (GPA, formerly known as Wegener’s granulomatosis) is a systemic necrotizing granulomatous vasculitis, often characterized by pulmonary symptoms in conjunction with renal dysfunction due to involvement of both systems [9]. Renal involvement is extremely common in the disease process, occurring in greater than 80% of reported cases of GPA [9]. Reported imaging features of GPA are variable, including renal scarring as well as solitary or even multiple renal masses [9, 10]. Like many vasculitides, GPA is highly responsive to steroids and cyclophosphamide [9] (Fig. 5).

### Leukocytoclastic vasculitis

**Fig. 6:**
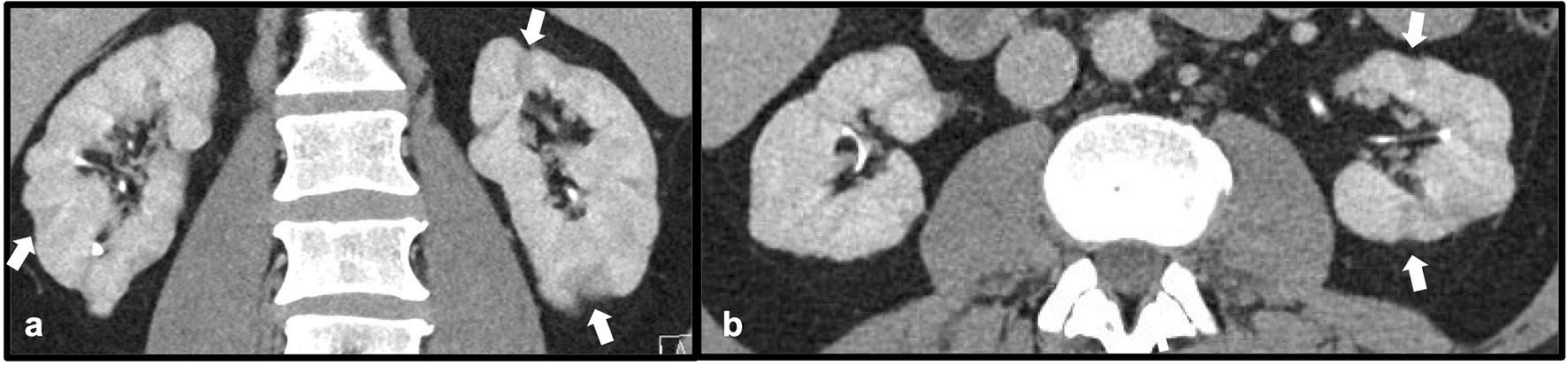
50-year-old male with biopsy-proven cutaneous LCV. Coronal (**a**) and axial (**b**) post-contrast CT images demonstrate multifocal peripheral wedge-shaped areas of renal cortical hypoattenuation and parenchymal loss bilaterally (white arrows) consistent with scarring. This patient had normal-appearing kidneys on a PET-CT 7 months prior

Leukocytoclastic vasculitis (LCV) is an inflammatory disorder involving post-capillary venules, generally thought to be the result of a hypersensitivity reaction [11, 12]. The disease can have manifestations in a wide variety of organ systems including the skin, mucous membranes, lungs, gastrointestinal tract and kidneys [11, 12]. Because cutaneous involvement is common, often presenting as palpable purpura, formal histopathologic diagnosis of LCV is often obtained via skin biopsy [12]. Renal involvement is common in LCV, with reported rates of necrotizing glomerulonephritis in up to 90% of cases [11]. Because the process involves small vessels, angiographic imaging may not directly demonstrate vascular complications such as aneurysms or pseudoaneurysms [11]. However, inflammatory changes within the involved organ system may be appreciated [11]. As with many vasculitides, LCV is commonly treated with steroids and immunosuppressant medications [12] (Fig. 6).

### Takayasu arteritis

**Fig. 7:**
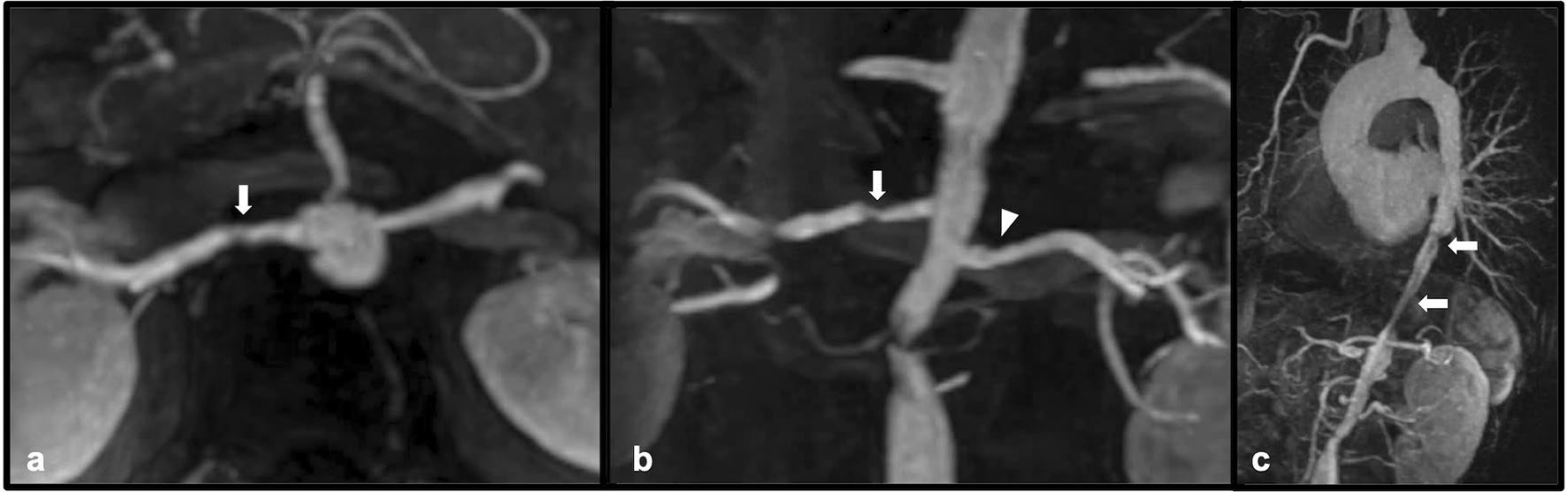
42-year-old female with known Takayasu arteritis. Axial (**a**) and coronal (**b**) post-contrast MR angiogram MIP images demonstrate focal severe stenosis (white arrows) of the proximal right renal artery. Mild irregularity of the proximal left renal artery (white arrowhead) is also present. Sagittal post-contrast subtracted MIP (**c**) demonstrates diffuse irregularity of the descending thoracic and abdominal aorta (white arrows)

**Fig. 8:**
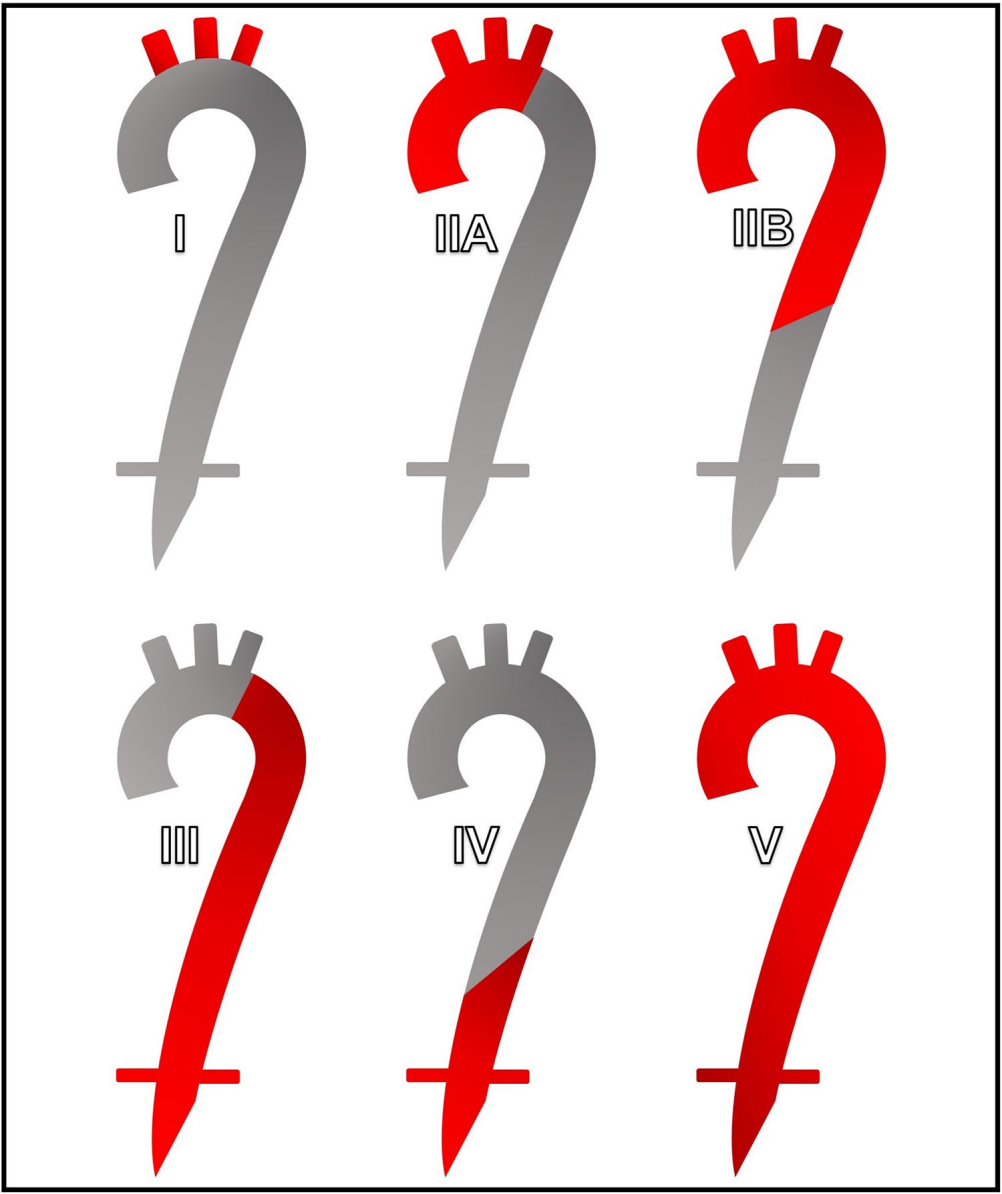
Graphic depicting the six subtypes of Takayasu arteritis. The red shading indicates areas of involvement. Note that the paired vessels at the inferior aspect of the illustrated aorta represent the renal arteries

Takayasu arteritis is a chronic inflammatory vasculitis that involves large arteries, often the aorta and its major branch vessels [13]. The disease is most commonly seen in young women in the second and third decade of life, and is classified into six subtypes, depending upon the sites of involvement, with types III – V involving the abdominal aorta and its branches with varying degrees of involvement of the thoracic aorta [13]. Renovascular involvement in Takayasu arteritis is a commonly reported feature, with CT findings often showing concentric mural thickening as well as luminal irregularity and narrowing which can contribute to significant stenosis [13]. Collateral vessels may also be seen as a result of hemodynamically significant stenosis [13]. Recognition of typical imaging features of Takayasu arteritis is essential, as appropriate treatment (such as corticosteroid administration) can help to prevent potentially catastrophic complications of the disease process, such as aneurysmal rupture and critical end-organ ischemia. In addition to management with corticosteroids, interventional management (such as percutaneous transluminal balloon angioplasty) and surgical bypass may be indicated in certain cases where hemodynamically significant stenosis is present [13] (Figs. 7 and 8).

### Immunoglobulin G4 vasculitis

**Fig. 9:**
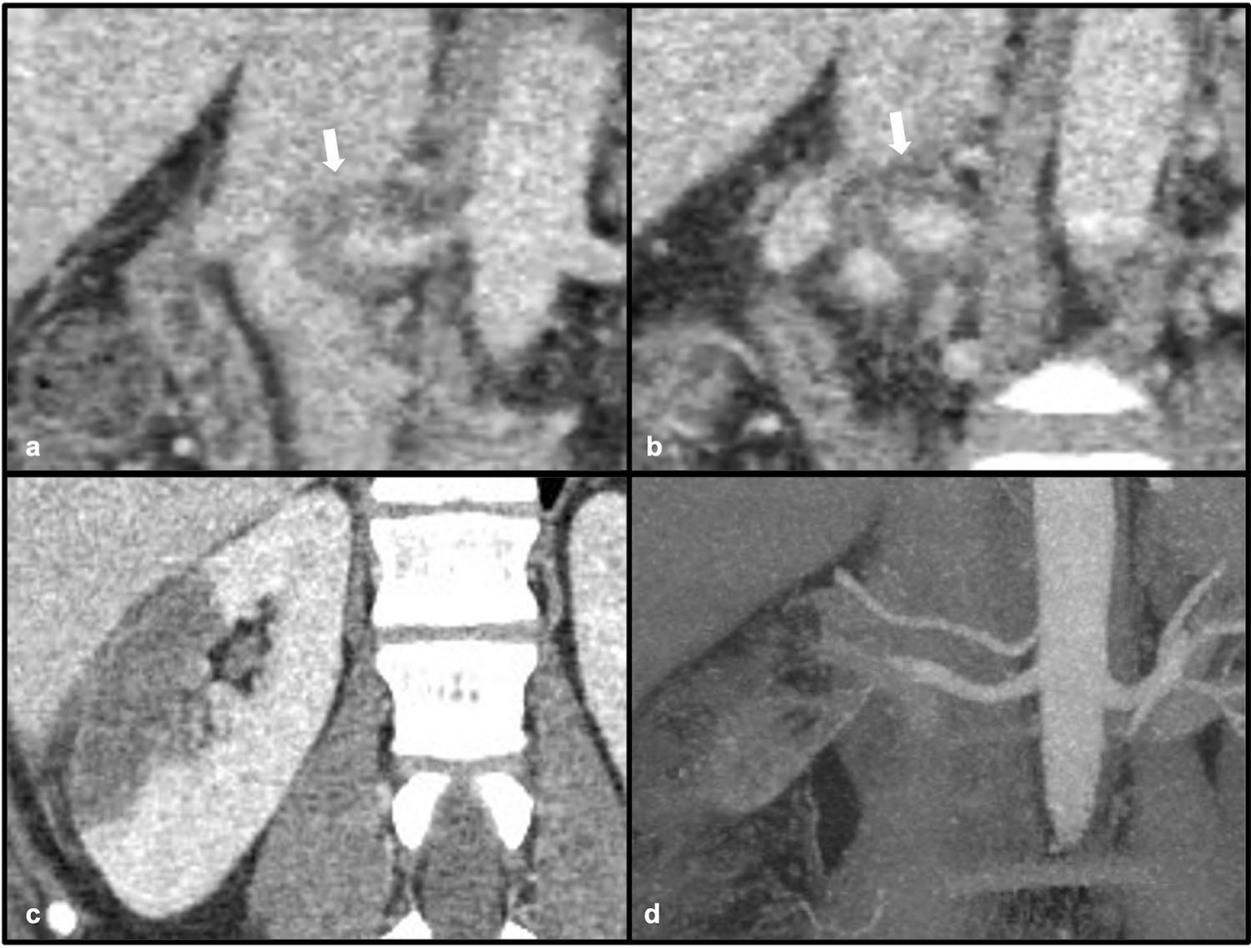
33-year-old male patient with confirmed IgG4 vasculitis. Coronal post-contrast CT images (**a**) and (**b**) demonstrate ill-defined, hypoenhancing soft tissue surrounding the right renal artery (white arrows) consistent with inflammation. Coronal post-contrast CT nephrogram (**c**) demonstrates a large, wedge-shaped cortical defect in the right kidney consistent with infarct. Coronal CT angiogram (**d**) shows resolution of the inflammation following steroid therapy

**Fig. 10:**
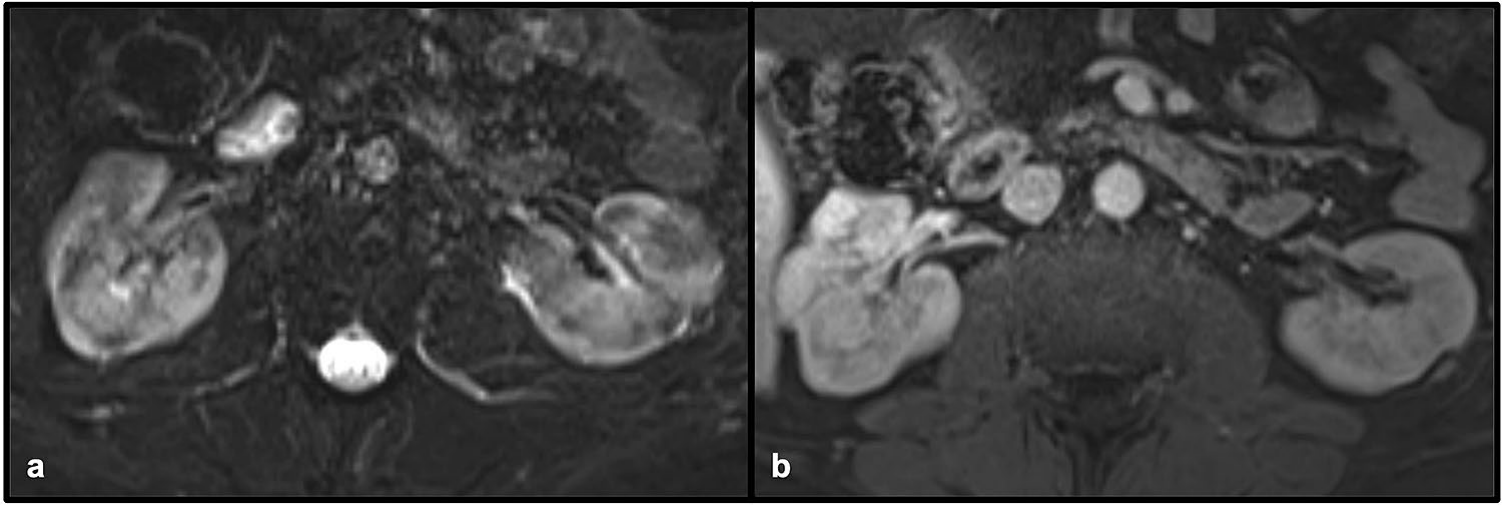
72-year-old male with acute kidney injury on a background of chronic kidney disease, with biopsy demonstrating IgG4 vasculitis. Axial T2 with fat saturation (**a**) and post-contrast GRE (**b**) demonstrate multifocal areas of renal cortical irregularity and scarring bilaterally

Immunoglobulin G4 (IgG4)-related disease is a multisystemic disease process related to infiltration of tissues by plasma cells and lymphocytes positive for IgG4, which can result in a variety of clinical manifestations [14]. The most well-known and classic association with IgG4-related disease is autoimmune pancreatitis, although additional sites of involvement (including renal involvement) are becoming more well recognized [14]. Renal involvement has been described in up to 35% of patients with autoimmune pancreatitis, and can have several different imaging manifestations, the most common being peripheral wedge-shaped cortical-based lesions, and less commonly a rim of tissue surrounding the kidney(s), diffuse patchy involvement, renal sinus nodules, and wall thickening of the renal pelvis [14]. Direct arterial involvement can also be seen with IgG4-related vasculitis with renal involvement. IgG4 disease is typically highly responsive to treatment with corticosteroids [14] (Figs. 9 and 10).

### Behçet’s disease

**Fig. 11:**
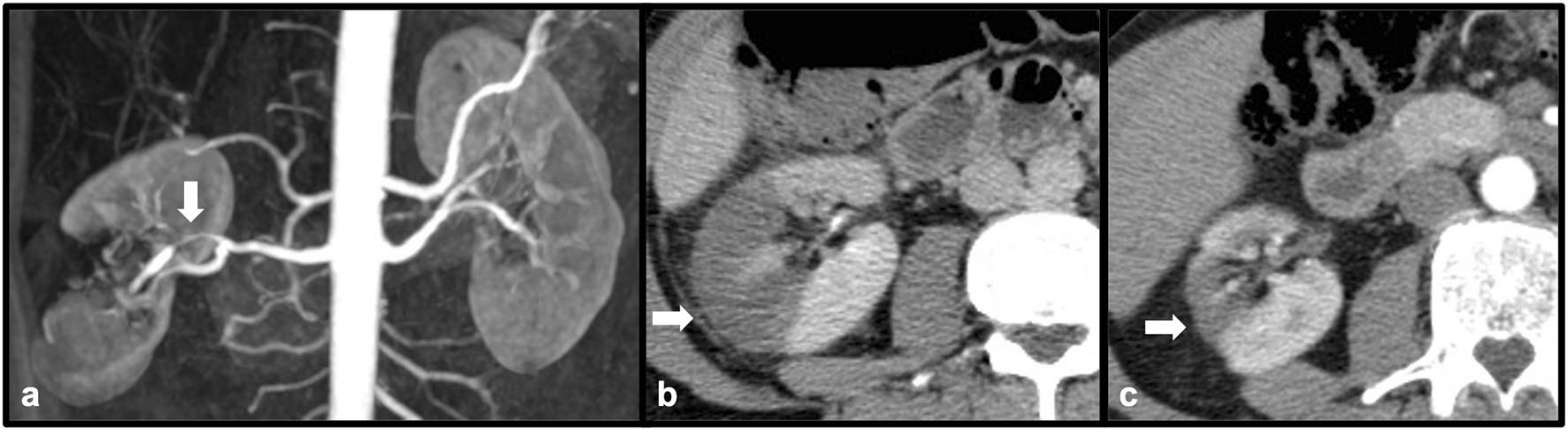
Coronal post-contrast MRI (**a**) in a patient with Behçet’s disease demonstrates narrowing of a branch of the main renal artery (arrow) with associated cortical defect consistent with infarct. Axial contrast-enhanced CT (**b**) demonstrates an area of diminished right renal perfusion (arrow) consistent with infarct. Axial contrast-enhanced CT 1 month later (**c**) demonstrates evolving infarct (arrow) in the right kidney

Behçet’s disease is a multisystem inflammatory vasculitis. While classically reported symptoms include oral and genital ulcers and uveitis, involvement is not confined to these areas, and multiple organ systems can be involved, including the venous and arterial systems, with the venous system being more commonly involved (accounting for approximately 85% of vascular involvement) [15]. Venous complications most commonly include thrombophlebitis, and less frequently venous occlusion and aneurysm formation [15]. Arterial vascular manifestations of Behçet’s disease include arterial aneurysms and pseudoaneurysms, thought to be the result of vasa vasorum inflammation resulting in elastic fiber destruction leading to vascular dilation [15, 16]. Additional arterial manifestations include stenosis and occlusion [16]. When encountered in the renal arterial system, areas of stenosis and occlusion can result in renal infarction. As with those of many of the vasculitides, manifestations of Behçet’s disease are often responsive to steroid therapy [16] (Fig. 11).

## Coagulopathic disorders

Many coagulopathic disorders can result in both arterial and venous thromboembolism, an important condition to recognize due to the potential for either inflow obstruction leading to arterial ischemia and infarction, or venous outflow obstruction leading to venous ischemia and organ dysfunction. While involvement of the renal vasculature may not necessarily reflect a specific entity, recognition of renal arterial or venous thrombosis could initiate a clinical coagulopathic workup which could ultimately guide therapy in these patients.

### Coronavirus disease 2019 (COVID-19)

**Fig. 12:**
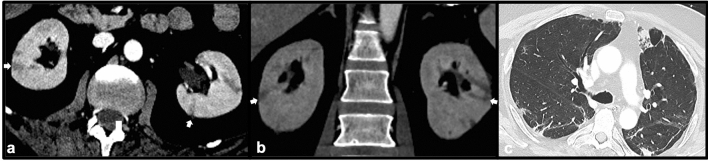
66-year-old male with COVID-19 infection and multiorgan failure requiring renal replacement therapy. Axial (**a**) and coronal (**b**) post-contrast CT images demonstrate multiple peripheral wedge-shaped cortical perfusion defects (white arrows) consistent with renal infarcts. Axial post-contrast CT in lung windows (**c**) demonstrates typical pulmonary findings of COVID-19 pneumonia

Infection with the SARS-CoV-2 Coronavirus, first identified in China in 2019, leads to the clinical syndrome of COVID-19, a complex infectious and inflammatory process that can be devastating to a variety of organ systems [17]. In addition to causing severe acute respiratory distress, patients with COVID-19 are at risk for multisystem organ dysfunction, including renal failure. In some instances, this may be attributable to thrombotic events, as patients with COVID-19 are at high risk for both arterial and venous thromboembolism [18]. These patients may present with abnormal renal function tests, oliguria or anuria, and elevated serum lactate dehydrogenase (LDH) levels during an episode of acute COVID-19 [18]. Contrast-enhanced CT may demonstrate wedge-shaped perfusion defects involving the renal cortex typical of renal infarction. Due to the highly pro-thrombotic nature of COVID-19 infection, patients are often placed on therapeutic anticoagulation to reduce the risk and severity of thrombotic complications [18] (Fig. 12).

### Nephrotic syndrome

**Fig. 13:**
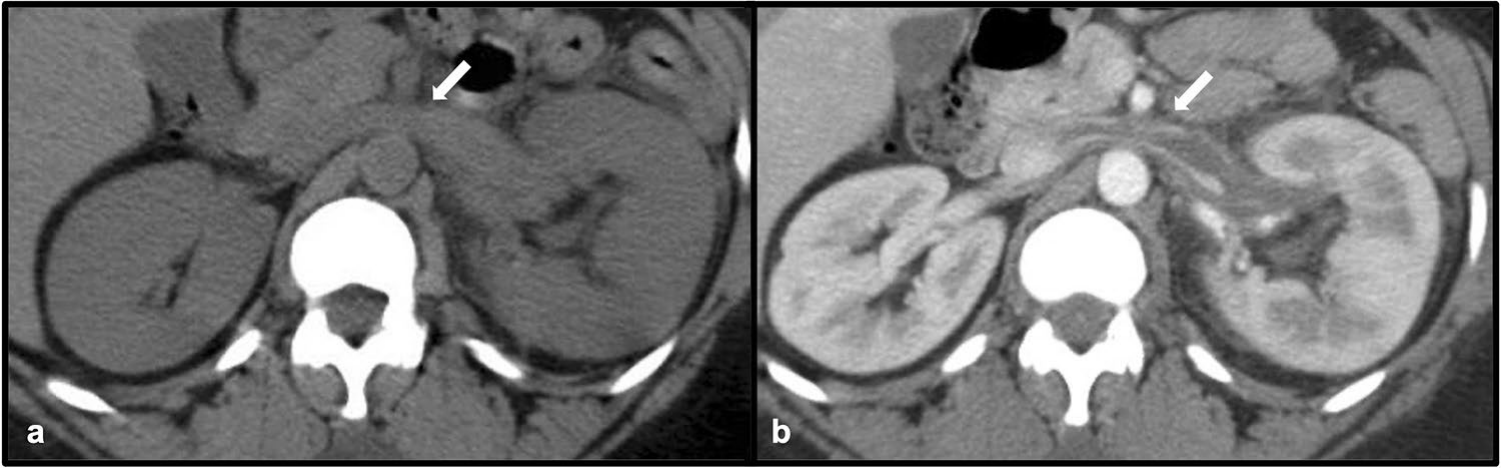
28-year-old male with nephrotic syndrome. Axial non-contrast CT of the abdomen (**a**) demonstrates expansion and hyperattenuation of the left renal vein (white arrow), as well as asymmetric enlargement of the left kidney. Axial contrast-enhanced CT (**b**) demonstrates a filling defect in the left renal vein consistent with renal vein thrombosis

Venous thromboembolism due to underlying hypercoagulability is a recognized phenomenon in nephrotic syndrome, possibly due to alterations in plasma coagulation protein levels, as well as increased platelet aggregation and treatment with steroids and diuretics [19]. Studies have demonstrated an incidence of symptomatic venous thromboembolism approaching 10% in patients with nephrotic syndrome within the first 6 months [19]. Most commonly, venous thromboembolic events in these patients are manifest by pulmonary emboli and/or deep vein thrombosis [19]. However, the incidence of renal vein thrombosis in patients with nephrotic syndrome has been found to be approximately 22%, indicating that the renal vasculature is susceptible to involvement in cases of nephrotic syndrome [19] (Fig. 13).

## Hemolytic diseases

Advances in MRI leading to excellent tissue contrast have allowed for localization of iron deposition in a variety of hematologic conditions, including primary and secondary hemochromatosis, systemic iron overload and hemolytic disease [20]. While iron deposition within the reticuloendothelial system (to include the liver, spleen and bone marrow) can indicate systemic iron overload, iron deposition within the renal cortex via the renal vasculature may provide additional details to elucidate the underlying etiology [20]. Therefore, characterization of intra-abdominal iron deposition patterns on MRI is key to understanding the underlying pathology.

### Mechanical intravascular hemolysis

**Fig. 14:**
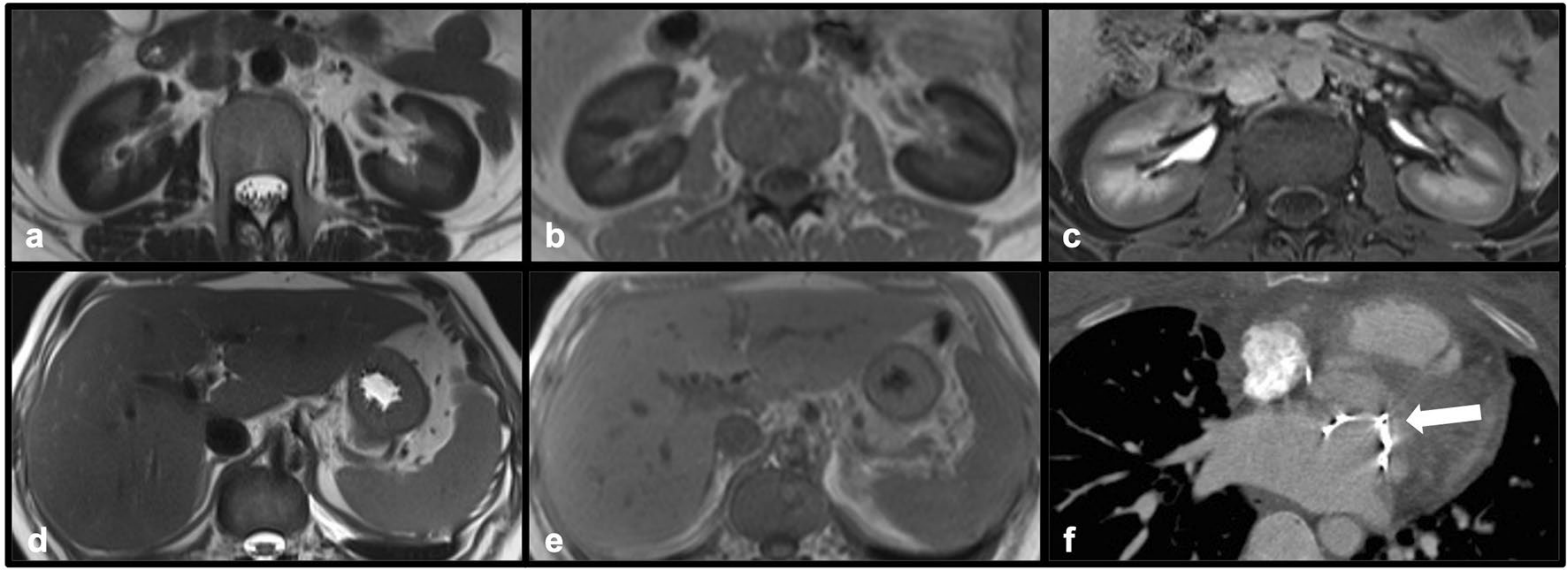
Axial T2 (**a**), GRE (**b**) and post-contrast T1-VIBE (**c**) MR images demonstrate diffusely decreased signal of the renal cortex bilaterally consistent with siderosis. Axial T2 (**d**) and GRE (**e**) demonstrate normal signal intensity of the liver and spleen. The prosthetic mitral valve (white arrow) resulting in hemolysis can be seen on axial contrast-enhanced CT (**f**), the source of renal cortical siderosis in this case

The presence of a mechanical prosthetic heart valve may lead to pure intravascular hemolysis. In these instances, deposition of iron in the renal cortex is often seen, best demonstrated on MRI. Due to the presence of hemosiderin within the proximal renal tubules, the renal cortex will demonstrate decreased signal intensity compared to the medulla on T1 weighted images, and significantly decreased cortical signal intensity will be observed on T2 weighted images [20]. Because hemolysis is purely intravascular in the setting of mechanical heart valves, abnormal signal intensity will not be present in the liver or spleen unless another underlying iron deposition pathology is present. In cases of mild hemolytic anemia related to mechanical cardiac valves, conservative therapy with beta blockers and iron supplementation can be considered, although severe cases may necessitate blood transfusion and even re-operation [21] (Fig. 14).

### Sickle cell disease

**Fig. 15:**
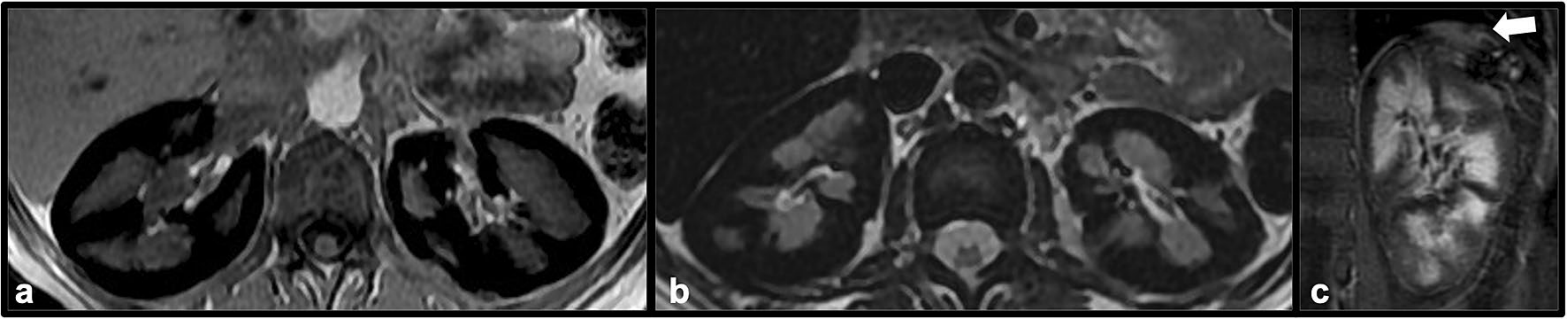
Axial GRE (**a**) and T2 (**b**) MR images in a patient with sickle cell disease demonstrate diffusely low renal cortical signal bilateral consistent with siderosis. The liver also demonstrates diffusely low T2 signal. Coronal post-contrast T1 image (**c**) demonstrates a small, largely infarcted spleen (white arrow)

Sickle cell disease results from production of an abnormal hemoglobin molecule (HbS) that results in abnormally shaped red blood cells resembling a “sickle”, which can result in hemolysis and vascular occlusion leading to ischemic and infarcted tissue in multiple organ systems [22]. Felt to be the result of intravascular hemolysis, renal cortical iron deposition has been found in patients with sickle cell disease, a feature that is well appreciated on MRI due to signal changes related to tissue iron content [23]. Using T2* weighted gradient echo sequences, the reciprocal T2* value (known as R2*) can be used to quantify iron content. Because sickle cell disease results in both intravascular and extravascular hemolysis, the liver and spleen are also characteristically low in T2 signal on MRI due to iron deposition, a feature not seen with pure intravascular hemolysis [23]. The spleen may be absent in cases of sickle cell disease, another important feature to identify when renal cortical siderosis is present. Management of sickle cell disease is complex but often relies on medications such as hydroxyurea to limit hemolysis and associated vaso-occlusive complications [24] (Fig. 15).

## Hematogenous spread

As in other organ systems, hematogenous processes may involve the renal vasculature and result in a variety of pathology. While hematologic spread of malignancy is outside the scope of this paper, additional processes including thromboembolism of both bland and infected embolic material can result in significant findings detectable by both CT and MRI. Some of these entities call for urgent interventional or surgical management, and therefore prompt recognition and conveyance to the clinician is necessary to optimize therapy.

### Thromboembolic disease

**Fig. 16:**
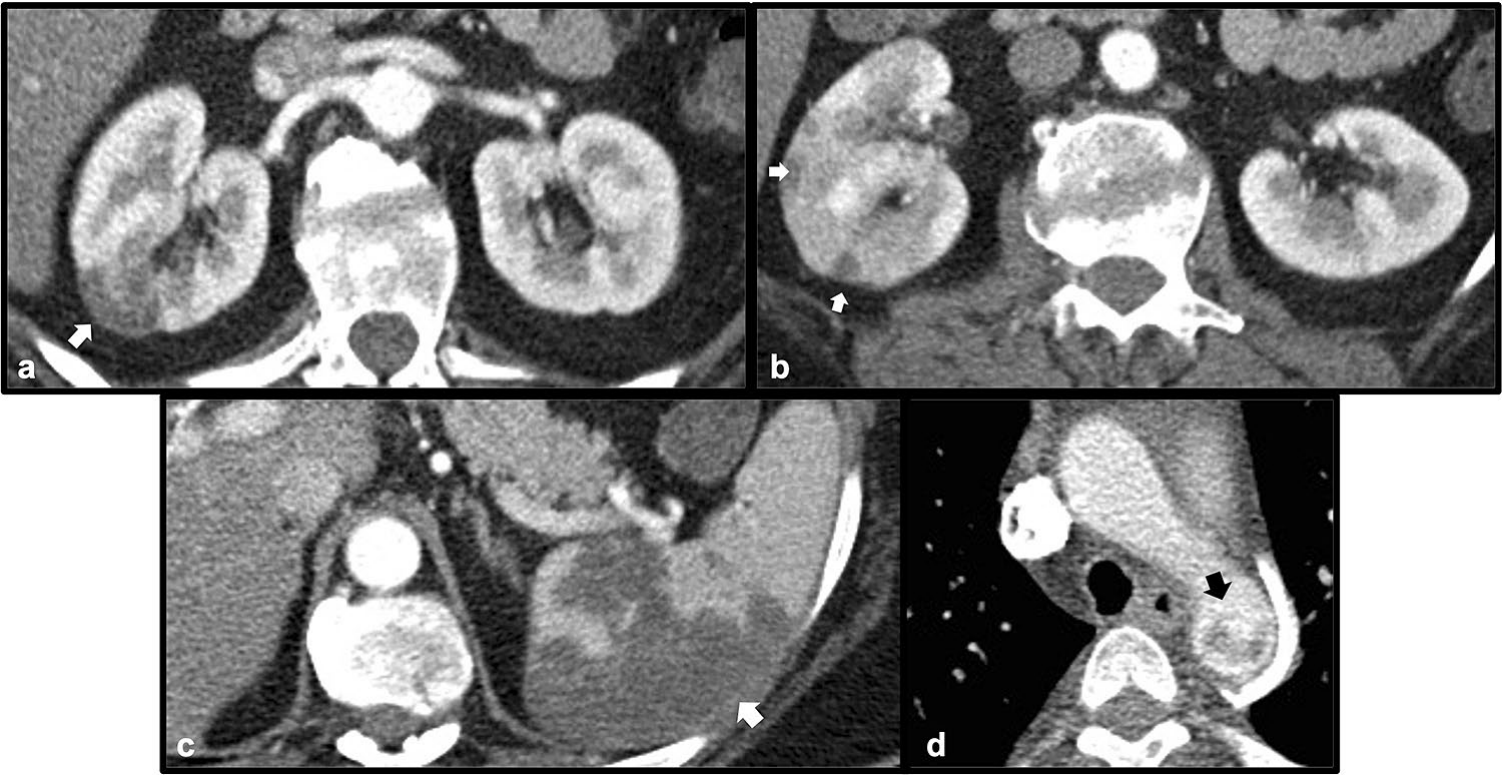
Axial post-contrast CT images (**a**-**b**) demonstrate multiple peripheral areas of low attenuation in the right kidney consistent with infarcts (white arrows). Axial post-contrast CT (**c**) demonstrates an additional infarct in the spleen (white arrow). A filling defect in the proximal descending thoracic aorta (black arrow) confirms mural aortic thrombus as the embolic source in this patient with atrial fibrillation

Acute occlusion of the renal artery is most commonly due to thromboembolism from an upstream site, usually the heart, with cardiac thrombus estimated to be the etiology of systemic renal artery embolism in approximately 94% of cases [25]. Renal artery occlusion may have a heterogeneous clinical presentation, depending upon the acuity of the occlusion and the degree of renal infarction, although common symptoms may include acute onset flank pain, hematuria, fever and leukocytosis, as well as elevated lactate dehydrogenase levels (LDH) in the setting of renal infarction [25]. Contrast-enhanced CT may demonstrate enlargement of the affected kidney as well as decreased enhancement due to diminished vascular perfusion. A filling defect in the renal artery on arterial-phase imaging may help to solidify the diagnosis and guide the clinicians towards the appropriate workup and therapy. Therapy for bland renal artery thromboembolism includes anticoagulation, as well as percutaneous thrombolysis or mechanical thrombectomy in some cases [26] (Fig. 16).

### Mycotic aneurysms

**Fig. 17:**
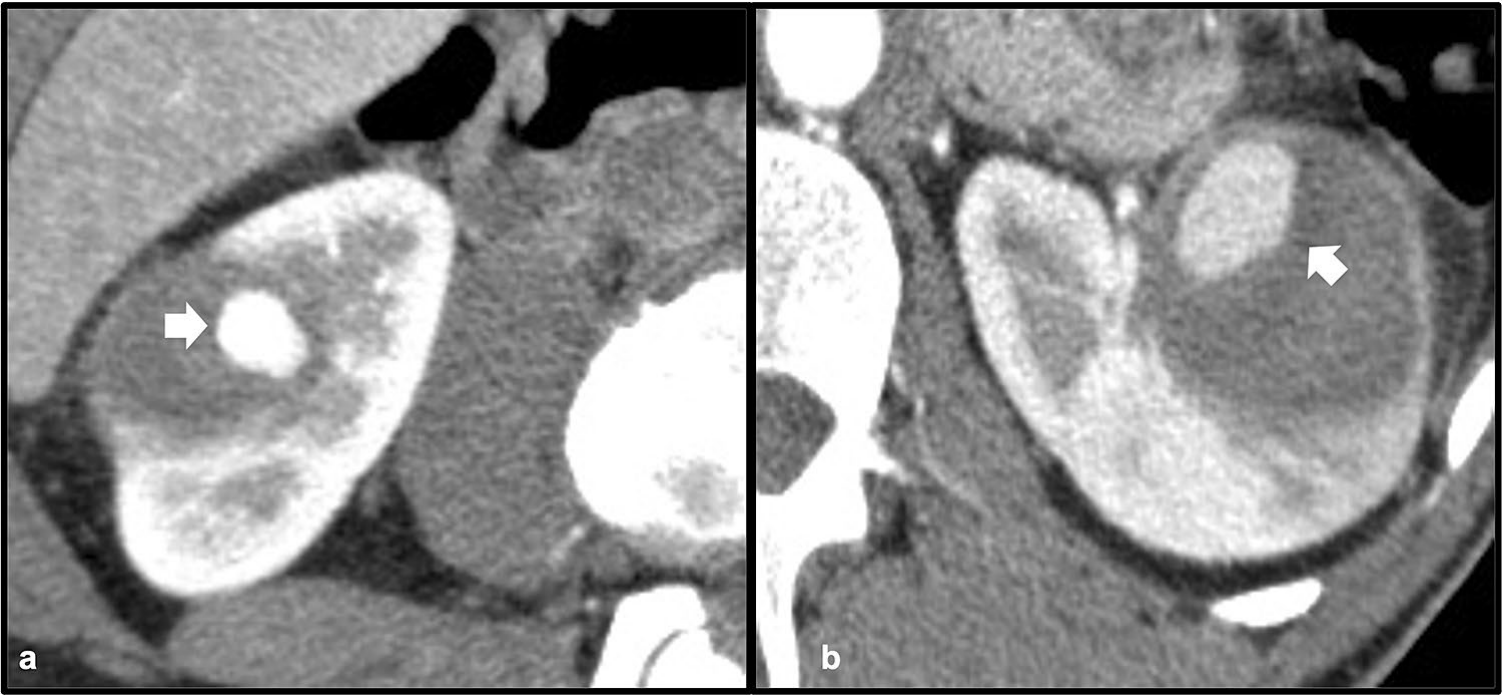
48-year-old male with a history of intravenous drug abuse who presented with flank pain and hematuria 3 weeks following coronary angiography and stenting. Axial post-contrast CT (**a**-**b**) demonstrates bilateral renal artery aneurysms (white arrows) consistent with intraparenchymal mycotic aneurysms. These were treated with embolization

Mycotic aneurysms are the result of infectious breakdown of a vessel wall leading to saccular collections arising from the arterial lumen [27]. These are most often found in intravenous drug users, immunosuppressed patients and following endovascular interventions, and are commonly caused by *Streptococcus* and *Staphylococcus* in the West [27]. The saccular collections are at high risk for rupture which can lead to massive hemorrhage and death, and therefore recognition of these entities is critical to prevent severe patient morbidity and mortality. The most common site of involvement is the aorta, although cerebral, peripheral and visceral arteries can be involved. Of the visceral arteries, the superior mesenteric artery (SMA) is most frequently involved, but involvement of arteries supplying other organs including the kidneys and spleen have been described [27]. CT angiography allows for excellent visualization of these saccular aneurysms and can serve as a valuable diagnostic and pre-surgical tool. Therapy for mycotic aneurysms involving the renal arteries includes antibiotics with either endovascular or open surgical repair of the aneurysm sac [28] (Fig. 17).

### Septic emboli

**Fig. 18:**
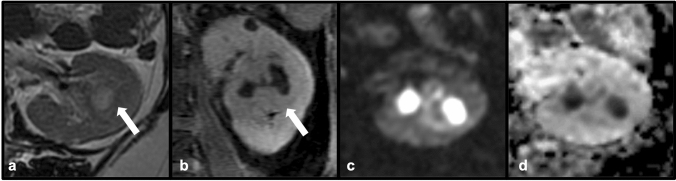
Axial T2 (**a**) and coronal post-contrast T1-LAVA (**b**) MRI demonstrates a T2 hyperintense, bilobed and thick-walled collection within the left kidney (white arrows). Increased signal on axial diffusion-weighted image (DWI) and corresponding decreased signal on absolute diffusion coefficient (ADC) map (**c** and **d**) consistent with diffusion restriction confirm the presence of a left renal abscess. This patient had known endocarditis suggesting septic embolism as the source

While the majority of renal abscesses result from the coalescence of smaller areas of suppuration into a larger collection, renal abscess may also develop in the setting of septic embolism to the kidney [29]. In patients with bacterial endocarditis, emboli to the kidneys may result in renal infarction or mycotic aneurysm formation [7, 27]. The presence of a thick-walled, complex intra-renal collection on CT in a patient with underlying bacteremia and endocarditis should raise concern for septic embolism. Correlation with clinical findings of endocarditis is therefore critical when renal abscess is identified, as it may help to clarify the underlying etiology and guide therapy. As with septic emboli within other organ systems, therapy consists of antibiotics and the addition of source control when technically feasible [30] (Fig. 18)

### Septic thrombophlebitis

**Fig. 19:**
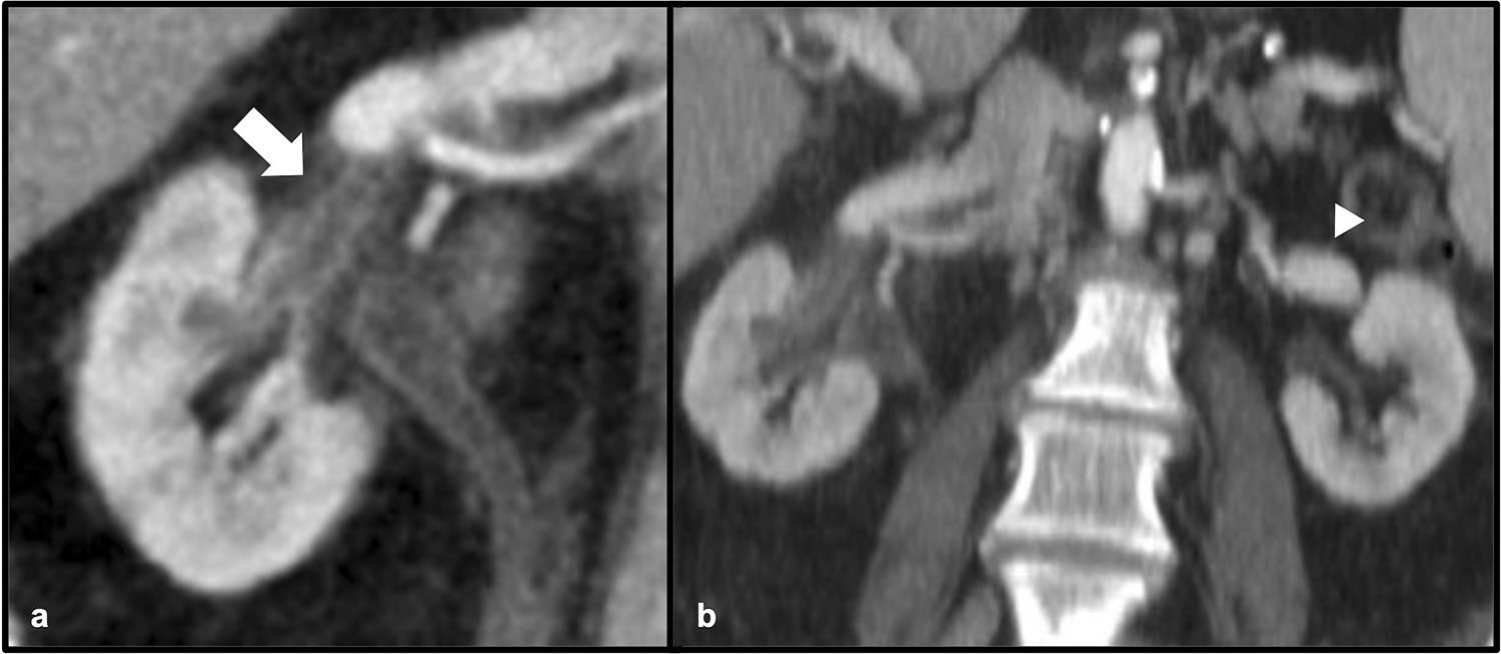
80-year-old male with a remote history of pulmonary squamous cell carcinoma status-post recent resection of benign pulmonary nodule who presented with fever, found to have methicillin-sensitive *Staphylococcus aureus* bacteremia. Coronal post-contrast CT (**a**) demonstrates a filling defect (white arrow) within a tributary of the right renal vein, with adjacent fat stranding, consistent with septic thrombophlebitis. Coronal post-contrast CT (**b**) demonstrates the extent of the right renal vein thrombosis. Note the normal-appearing left renal vein (white arrowhead) for comparison

Septic thrombophlebitis describes the formation of vascular thrombus as a result of adjacent bacterial infection. While the exact mechanism of thrombus formation is not entirely understood, invasion of adjacent pathogens into the vein with resultant thrombus formation has been suggested, with possible implication of certain bacterial endotoxins resulting in disruption of normal vascular endothelial function [31, 32]. Septic thrombophlebitis may occur in superficial veins, dural venous sinuses, portal and pelvic veins [31]. Involvement of the renal veins and inferior vena cava have also been described in the setting of pyelonephritis [32]. The presence of a renal vein filling defect in the setting of sepsis or bacteremia should prompt consideration of septic thrombophlebitis. Antibiotics and anticoagulation are the primary therapy for visceral septic thrombophlebitis [31] (Fig. 19).

## Miscellaneous conditions

Several entities, including fibromuscular dysplasia and hereditary hemorrhagic telangiectasia, constitute non-vasculitic systemic disease processes that may involve the renal vasculature. These entities often demonstrate characteristic features that are detectable on CT and MRI, and therefore recognition of these typical disease patterns can lead to the correct diagnosis and allow for the initiation of appropriate therapy. Because these diseases are non-inflammatory in nature, distinguishing them from the vasculitides is crucial, as therapy may differ significantly.

### Fibromuscular dysplasia

**Fig. 20:**
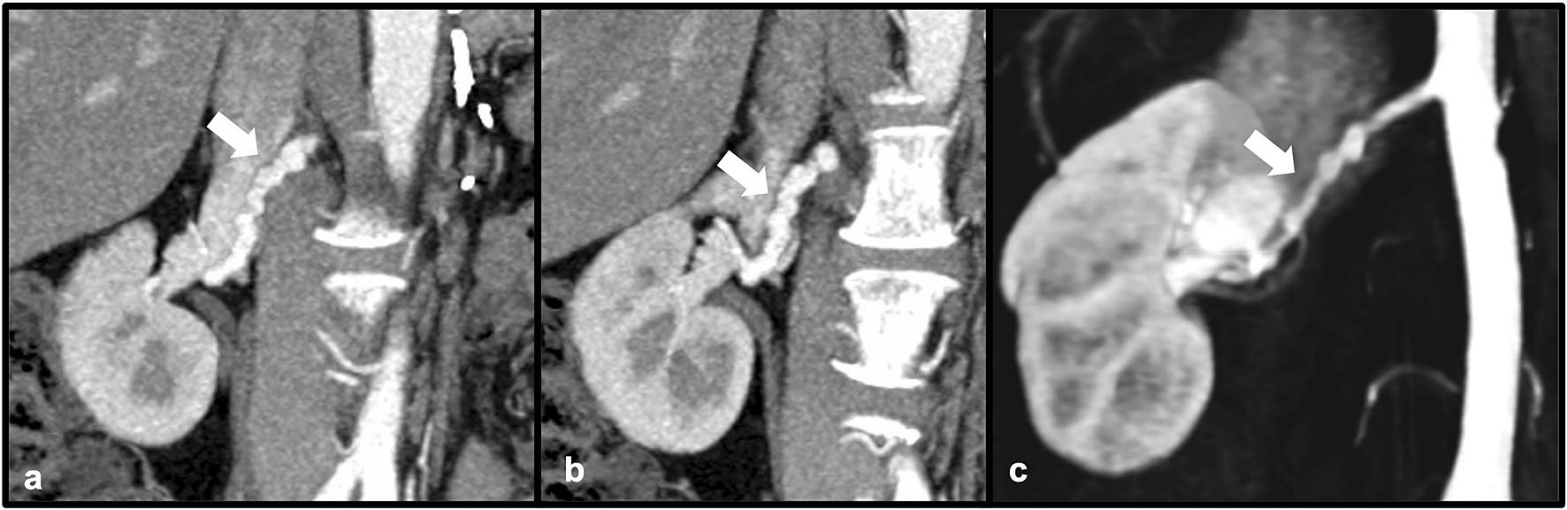
52-year-old male with oliguria and abnormal renal function. Coronal contrast-enhanced CT (**a** and **b**) demonstrate beading and irregularity of the right main renal artery (arrows). Coronal post-contrast MRI (**c**) also demonstrates beading and irregularity of the right renal artery (arrow) consistent with FMD

**Fig. 21:**
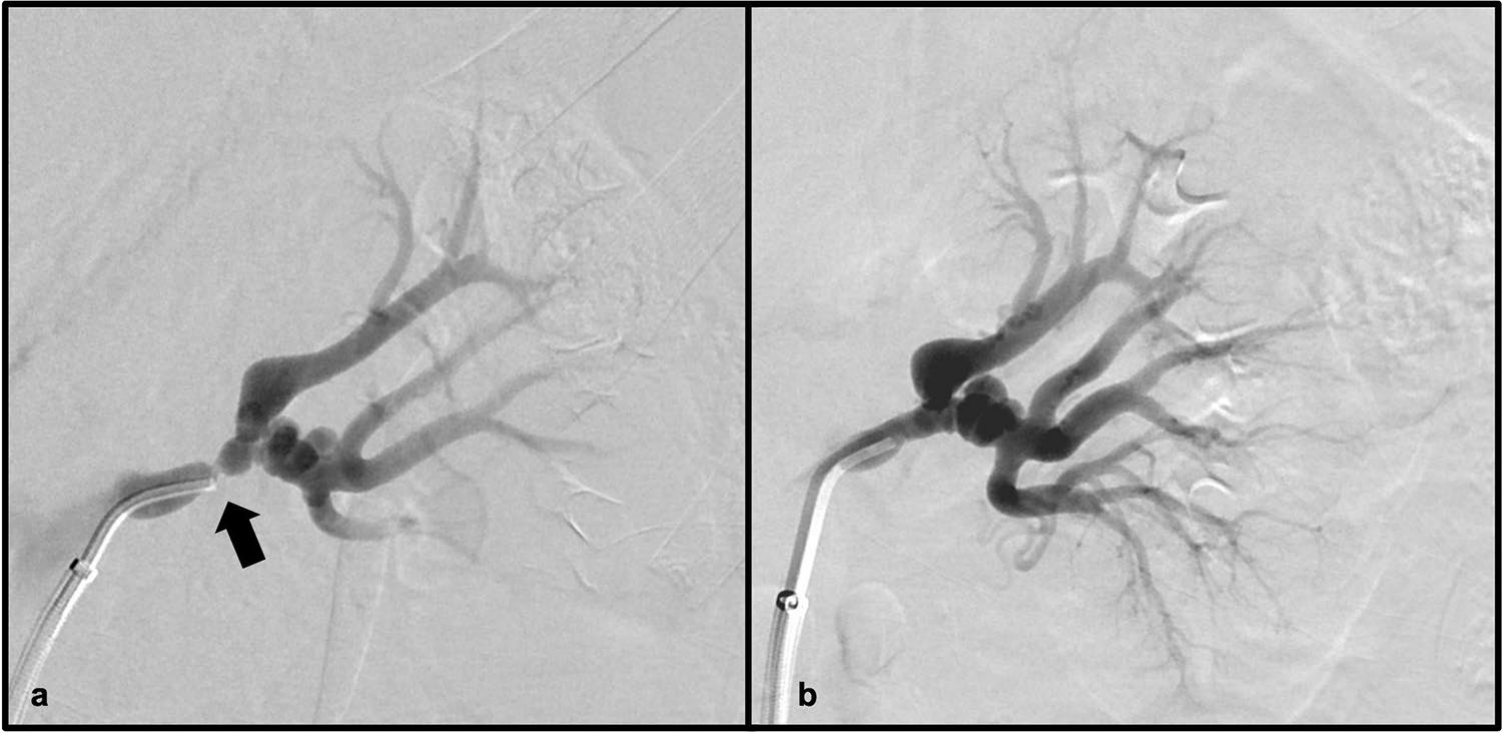
20-year-old male with uncontrolled hypertension since age 16, found to have FMD. Pre-intervention angiography (**a**) demonstrates irregularity of the left renal artery with a focal area of high-grade stenosis (arrow). Post-angioplasty angiogram (**b**) demonstrates marked improvement of the focal stenosis

Fibromuscular dysplasia (FMD) is an idiopathic, non-inflammatory process involving small and medium-sized arteries, of which the etiology is not well understood [33]. Multiple histopathologic subtypes exist, depending upon the location of fibrous proliferation and elastic deposition (intimal, medial, perimedial/subadventitial), with the medial type being the most common [33]. Prevalence of FMD involving the renal vasculature is estimated between 4 and 6%, although this may be underestimated when accounting for asymptomatic cases [33]. The multifocal “string of beads” lesion is most common, occurring in up to 84% of patients with symptomatic renal FMD, although additional imaging features including arterial dissection and aneurysm have been reported [33]. Bilateral involvement of the renal arteries is also common, occurring in up to 54% of symptomatic patients [33]. Because renal involvement in FMD frequently leads to renovascular hypertension, therapy consists of medical anti-hypertensive therapy and percutaneous transluminal balloon angioplasty, with surgical management reserved for severe or refractory cases [34] (Figs. 20 and 21).

### Hereditary hemorrhagic telangiectasia

**Fig. 22:**
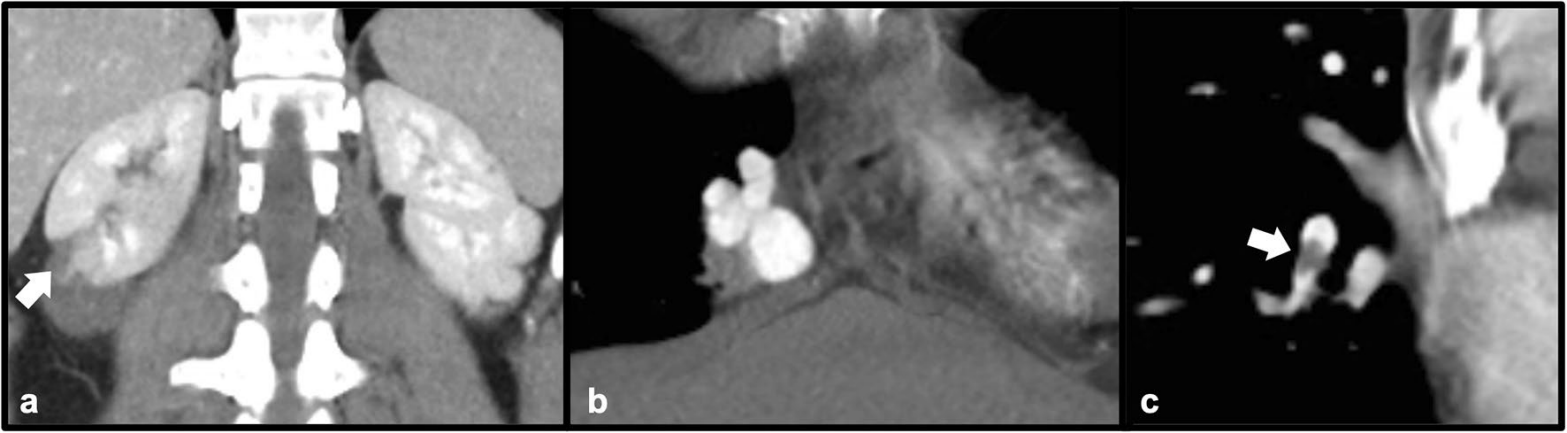
42-year-old female with right lower quadrant abdominal pain. Coronal contrast-enhanced CT (**a**) demonstrates a wedge-shaped cortical defect in the right inferior renal pole (white arrow) consistent with renal infarct. Coronal contrast-enhanced CT pulmonary angiogram (**b** and **c**) demonstrates an enlarged, tortuous pulmonary artery with pulmonary venous communication consistent with pulmonary AVM. A filling defect (white arrow) can be seen within a pulmonary artery supplying the AVM consistent with pulmonary embolism, with probable embolic fragments passing through the AVM due to right-to-left shunting, leading to embolic renal infarction

Hereditary hemorrhagic telangiectasia (HHT), also known as Osler-Weber-Rendu disease, is an autosomal dominant hereditary vascular disease typically manifest by the presence of multiple arteriovenous communications throughout the body [35]. Classically, affected systems include the lungs, resulting in pulmonary arteriovenous malformations (AVMs), as well as the skin and mucous membranes. However, telangiectasis, arteriovenous malformations and shunts may be found in multiple additional organs such as the liver, pancreas and large intestine [35]. Renal arteriovenous malformations have also been found in patients with HHT [36]. Because of arteriovenous shunting, these patients may be at risk for high-output cardiac failure and other complications (including right-to-left embolic complications), and therefore recognition of these shunts is important to help guide endovascular and other therapies [35] (Fig. 22)

## Conclusions

A wide range of systemic vascular disease processes may involve the renal vasculature, resulting in a variety of imaging appearances, which can be characteristic in the correct clinical setting. Both vasculitic and non-vasculitic renovascular diseases may result in potentially significant or even life-threatening complications, and therefore recognition of the imaging findings related to these processes is essential. Advances in CT and MR imaging techniques have allowed for excellent visualization of renal vasculature and parenchyma, which can provide clues for the formation of a diagnosis that ultimately helps guide therapy for these entities. Therefore, recognition of renovascular involvement of systemic vascular conditions at non-invasive imaging remains a fundamental component of caring for patients with these diseases.
